# Decline of salt marsh-nesting birds within the lower Chesapeake Bay (1992–2021)

**DOI:** 10.1371/journal.pone.0323254

**Published:** 2025-06-02

**Authors:** Bryan D. Watts

**Affiliations:** Center for Conservation Biology, William and Mary, Williamsburg, Virginia, United States of America; Southeastern Louisiana University, UNITED STATES OF AMERICA

## Abstract

Bird species that depend on tidal marshes throughout the world are threatened by ongoing sea-level rise. How species that differ in their level of marsh-dependency may respond to change over time remains unclear. I surveyed a network of patches (N = 186) within tidal salt marshes located in the lower Chesapeake Bay (1992, 2021) for 12 species of breeding birds to evaluate changes in abundance. Marsh-nesting bird abundance declined by 65.7% during the course of the survey interval. Significant declines in abundance were discovered for eight of ten species evaluated with declines in abundance ranging from 34 to 100%. Four species were extirpated or nearly extirpated within focal marshes during the study period. The magnitude of decline was highest for facultative nesting species (84.2%) followed by marsh obligates (81.6%) and salt marsh obligates (44.2%) respectively. Salt marsh obligates have become an increasingly dominant portion of the species assemblage over time reaching 83% of all detections by 2021. This pattern supports the prediction that specialists may persist longer than generalists as habitats are subjected to change. Despite their relative stability, salt marsh obligates are of high conservation concern over the longer term due to their specialization on a habitat that is currently experiencing rapid disruption. Even though this study did not evaluate the causes of population decline, results are aligned with other recent work within other regions that have implicated ongoing sea-level rise and nest predation.

## Introduction

Tidal wetlands and the unique wildlife value they provide are under increasing threat as sea-level rise accelerates. The complex processes that allow tidal marshes to keep pace with rising seas have physical and biological limits [1] that are being exceeded within many areas throughout the globe primarily within human-dominated landscapes where transgression is restricted and marshes are sediment starved [2]. Even within geographic areas where marshes are capable of maintaining their physical structure, increases in the frequency, severity and duration of flooding events associated with sea-level rise may cause shifts in plant composition [3] and demographic stress for species that depend on them for breeding [4,5]. Along the Atlantic Coast of North America, these habitats support a complex of endemic nesting birds that are adapted to tidal life [6,7]. Several of these unique forms are now declining [e.g., 8–10] or threatened with extirpation on regional to range-wide scales [e.g., 11,5,12,13].

The Chesapeake Bay is the largest estuary in North America and supports an extensive system of tidal wetlands [14]. The Bay is experiencing one of the highest rates of sea-level rise in the United States [15]. Relative sea-level rise (3–6 mm year^-1^) is twice the global rate due to subsidence (1.6–2.0 mm year^-1^) [16]). Sea-level rise within the Bay has accelerated from 1 to 3 mm year^-1^ in the 1930s to 4–10 mm year^-1^ in 2011 [17,18]. Vertical accretion of marshes within some geographic areas of the Bay is not keeping pace with sea-level rise [19]. However, over the past one hundred years tidal wetland area has remained stable as the rates of conversion of wetlands to open water and conversion of uplands to wetlands have been in balance [20]. To date this dynamic balance has been maintained due to the relationship between sea-level rise and the relatively low slope of uplands surrounding marshes. Whether or not the increased flooding regime often associated with sea-level rise has allowed wildlife populations to keep pace with marsh area is unknown.

Tidal salt marshes within the Bay provide breeding habitat for an assemblage of birds that vary in their level of specialization on this habitat from obligate to facultative [21–23]. Obligate salt marsh nesters are those species that breed only within the salt marsh. Obligate marsh nesters are those species that nest in salt marshes but also nest in other tidal or nontidal wetlands. Facultative marsh nesters are those species that nest in salt marshes but also nest in upland habitats. Although the marsh-nesting bird community within the Chesapeake Bay has been shown to be impacted by the proximity and extent of human development [24], we know very little about trends for marsh-nesting populations or the influence of specialization on trends.

The degree of specialization in marsh-nesting birds has been shown to be associated with a short-term advantage but a longer-term disadvantage in terms of regional population trends in the face of sea-level rise [25]. Specialists that are adapted to tidal life are presumably more resilient to changes in tidal variation and flooding events compared to generalists. However, as marshes continue to be degraded (e.g., erosion, vegetational shifts), specialists are effectively trapped within their singular habitat while generalists are free to breed within alternate habitats. These contrasting short and long-term advantages suggest that specialists may persist longer than generalists within marshes but will ultimately go extinct as marshes disappear or become uninhabitable. In this study, I resurvey nesting birds in 2021 within a set of reference salt marshes in the lower Chesapeake Bay that were originally established and surveyed in 1992 to evaluate potential changes in occupancy and abundance. I evaluate changes for individual species and groups of species according to degree of specialization.

## Methods

### Study area

**Fig 1:**
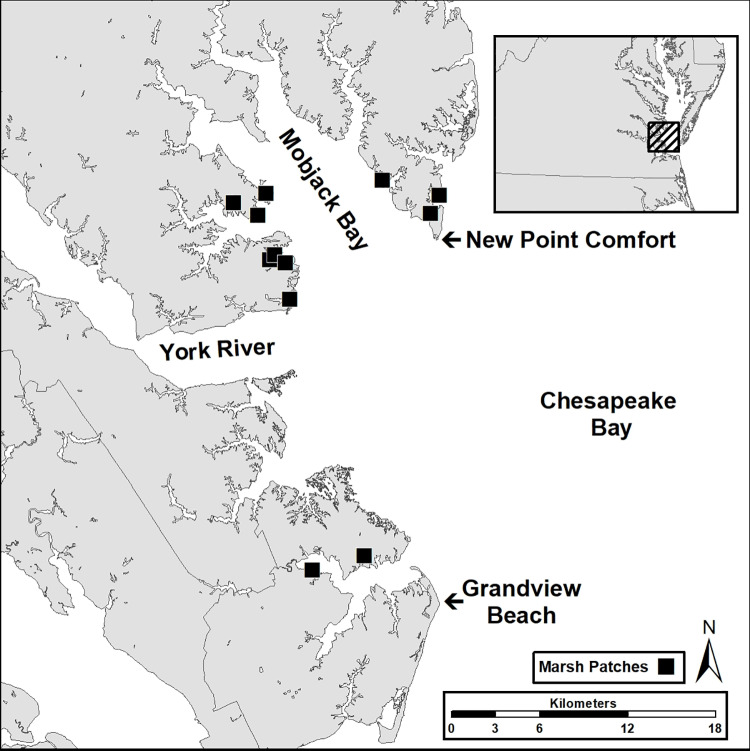
Map of the study area within the Chesapeake Bay of Virginia where tidal marsh nesting birds were surveyed (1992, 2021). Dark squares indicate the location of focal marshes included within the study.

I sampled marsh birds along the western shore of the lower Chesapeake Bay between Grandview Beach (City of Hampton) and New Point Comfort (Mathews County) in Virginia (Fig 1). The broad land arc within these boundaries supports some of the most extensive tidal marshes remaining in the lower Bay including over 1,300 marsh patches with a total area of 6,200 ha (https://www.vims.edu/ccrm/research/inventory/virginia/index.php). Marshes within the area are polyhaline (18–30 ppt) with a vegetation community dominated by smooth cordgrass (*Spartina alterniflora*), black needlerush (*Juncus roemerianus*), salt meadow hay (*Spartina patens*), salt grass (*Distichilis spicata*) and groundsel tree (*Baccharis halimifolia*) [26–29]. Breeding species of marsh birds within the study area are area sensitive [22,30]. The marsh obligate community found within large (>65 ha) patches collapses as patch size is reduced from 5 to 1 ha with none of the species occurring in marsh patches < 1 ha.

### Bird surveys

I selected four replicate marsh patches within the size categories 5 ha (actual mean size = 5.5 ha ± 0.12 standard error), 10 ha (9.9 ± 0.33) and >65 ha (98.7 ± 29.36) to represent the bird community within the study area. I used marsh inventories for the study area [27–29] that were available in 1992 to screen marshes for possible inclusion within the study. Patch size (categories above), vegetation composition and accessibility (accessible from uplands or within 2 km by boat) were used as criteria for selection. Patches were considered for inclusion if they were dominated (>90%) by and contained all of the plant forms including smooth cordgrass (≥30%), black needlerush (≥20%), salt meadow hay and salt grass combined (≥15%) and groundsel tree (≥10%). Replicate patches were randomly selected for each size category from a pool of marshes that met criteria. Marsh patches included in the study were owned by the United States Department of Defense, United States Fish and Wildlife Service and many private landowners. Permission to access properties for surveys was obtained from the ownership agencies and all private landowners.

The set of marsh patches included in this study are not a random sample of those available. The majority (55.4%) of marsh patches (N = 1,295) within the study area are < 1 ha in area with 81.9% falling below 5 ha. However, despite the low availability of larger (> 5 ha) marshes, the large marshes collectively account for 81.7% of the total marsh area available within the study area. Because marsh birds are sensitive to patch area, size categories (5, 10, > 65 ha) were selected that were above the known inflection point for the community [22,30].

I surveyed birds within marsh patches using the same network of 30-m, fixed-radius plots (n = 186) during two time periods (1992, 2021). A series of transects was established within each patch that extended up the elevational gradient of the marsh from open water to the marsh-upland ecotone. This structure was used to ensure that all vegetation types were sampled within each patch. Transects were separated by a minimum of 70 m. Centers of survey plots were positioned along transects and were separated by a minimum of 70 m. The percentage of the marsh surface that was included within survey plots declined with increasing patch size (5 ha: 45.0 ± 5.69%, 10 ha: 31.9 ± 1.19%, > 65 ha: 9.5 ± 1.82%).

I surveyed birds within 30-m fixed radius plots using a call-response technique [31] to increase rail detections. The call-response survey consisted of a 5-min sequence of alternating silent listening periods and species recordings in the following order; 1) 30 sec of silence; 2) 50 sec of black rail advertising call; 3) 10 sec of silence; 4) 50 sec of Virginia rail advertising call; 5) 10 sec of silence; 6) 50 sec of clapper rail advertising call; 7) 10 sec of silence; 8) 50 sec of king rail advertising call; 9) 40 sec of silence. Although black and king rails were never detected during either survey period, the playback sequence was maintained for consistency. I executed counts by standing at the plot center and recording birds seen or heard during the playback sequence. No permits were required for this work since the study only included observational data. In 1992 the plot center was marked with a numbered wire flag and the perimeter of the plot was marked in the four cardinal directions with flagging tape to facilitate the determination if birds were within the plot circle. In 2021 the plot center was marked with numbered flagging tape and a digital rangefinder (Nikon ProStaff Laser440) was used to determine if birds detected were within the plot. Survey techniques for marsh birds have evolved over time toward variable-radius and time-segmented approaches with the eventual establishment of a more standardized approach [32]. The survey approach used here in 1992 was utilized in 2021 for comparative purposes.

Surveys were conducted between sunrise and 4 hours after sunrise. All marshes were surveyed four times during each year. To reduce seasonal bias and ensure even coverage, marshes were surveyed in four rounds where all marshes were surveyed in each round and the survey order was randomly determined. A split survey approach was used to accommodate differences in calling behavior and residency among target bird species, with the first two rounds conducted between 6 May and 3 June to capture peak vocal activity for rails, while the later rounds were conducted between 11 June and 10 July to detect species with later breeding schedules.

Species that breed in saltmarshes have a range of affinities for this habitat type within the study area [21,22,30]. Obligate saltmarsh nesters are those species that breed only within the saltmarsh including American black duck (*Anas rubripes*), clapper rail (*Rallus crepitans*), willet (*Tringa semipalmata*), saltmarsh sparrow (*Ammodramus caudacutus*) and seaside sparrow (*A. maritimus*). Obligate marsh nesters are those species that nest in saltmarshes but also nest in other tidal or nontidal wetlands including Virginia rail (*R. limicola*), sedge wren (*Cistothorus platensis*), marsh wren (*C. palustris*) and red-winged blackbird (*Agelaius phoeniceus*). Facultative marsh nesters are those species that nest in saltmarshes but also nest in upland habitats including song sparrow (*melospiza melodia*) boat-tailed grackle (*Quiscalus major*) and eastern meadowlark (*Sturnella magna*). All species were included within the following analyses except for American black duck and saltmarsh sparrow. American black ducks were detected within two marshes during 1992 surveys but not on survey plots and were not detected in study marshes during 2021. Saltmarsh sparrow is a winter resident and migrant within the study area and migrants do not entirely vacate the study area until early June. This species was detected within one survey plot during the later survey rounds in 1992 but not in 2021. A nest found during the 1992 surveys [33] may have been one of the last within the study area and the population is believed to have gone locally extinct between 1992 and 2021.

Other than using marsh inventory data to select marsh patches for inclusion in the study, no attempt was made to quantify vegetational composition or structure within survey plots. However, substrate use was recorded for individual observations during both 1992 and 2021. Statements made regarding substrate use should be considered observational rather than quantified results.

### Data analysis

I used the package ‘unmarked’ [34] in program R 4.4.2 [35] to construct N-mixture models that allow for state changes between sampling periods [36–38]. N-mixture models were constructed for each species individually, each group of birds (saltmarsh obligate, marsh obligate, and facultative), and all birds combined. I used year (1992 and 2021) as primary sampling periods and surveys conducted within each year (rounds 1–4) as secondary sampling periods. To evaluate relative fit for competing models, I used Akaike’s Information Criterion (AIC) for N-mixture analyses [39]. I selected the model with the lowest AIC as the best supported for each species when all other models had a ΔAIC > 2.0 [40]. If one or more models had a ΔAIC < 2.0 and the models were nested with similar log-likelihoods, I chose the more parsimonious model (i.e., fewest number of parameters) over more complex models [41,42].

I evaluated whether a Poisson or zero-inflated Poisson distribution better fit a model with zero predictors (i.e., null model) and then I compared the null model with a model including day of year in the detection function, a model including marsh area (continuous variable) in the abundance function and models that included both day of year in the detection function and marsh size in the abundance or occupancy function. I did not include predictors in the recruitment and apparent survival for N-mixture models. While the N-mixture models estimate recruitment and apparent survival, these metrics reflect the persistence of populations rather than individuals and serve as proxies for population stability.

I estimated posterior distributions for species-specific abundance using empirical Bayes methods for each final model. I extracted the mode of the abundance distribution for each survey point during each survey period (1992 and 2021) and generated standard errors from 1,000 iterations of non-parametric bootstrapping. I used Welch’s t-tests to compare species-specific abundance at each point between sampling periods (1992 and 2021) using survey plots as samples. For community-wide and group-specific data (obligate saltmarsh, obligate marsh, and facultative marsh nesters), I summed species-specific abundance at each point, calculated means and standard error, and then used Welch’s t-test to compare abundance between survey periods.

## Results

I conducted 1,480 surveys of plots (n = 186) during the two time periods (1992, 2021) and recorded 3,069 detections of target marsh-nesting species. Total raw detections were dominated (72.1%) by three obligate salt marsh species followed by four obligate marsh species (17.9%) and three facultative marsh species (10.0%). The seaside sparrow was the most abundant species accounting for 48.5% of all detections followed by clapper rail (13.0%) and willet (10.7%). The composition of the assemblage shifted over time toward salt marsh obligates with salt marsh obligate, marsh obligate and facultative marsh species accounting for 65.8%, 19.7% and 14.5% of detections respectively in 1992 but 83.1%, 14.9% and 2.0% of detections respectively in 2021. By 2021 seaside sparrows and clapper rails combined accounted for 74.9% of all detections.

**Fig 2:**
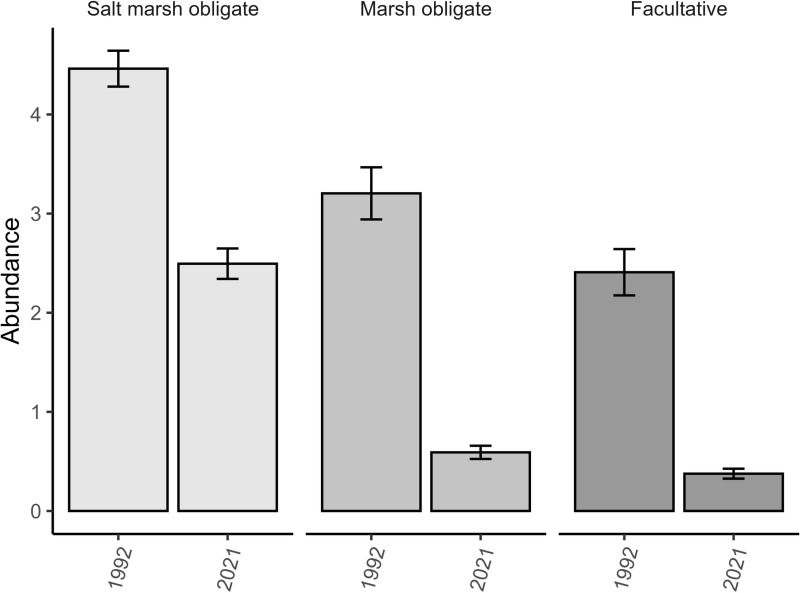
Abundance patterns for obligate salt marsh species, obligate marsh species and facultative marsh species surveyed within the lower Chesapeake Bay (1992, 2021). Bars represent mean values ± 1 standard error unit.

Community-wide and group abundance patterns show declines by all groups but higher declines for facultative marsh nesters and obligate marsh nesters compared to salt marsh nesters (Fig 2). The overall abundance of marsh-nesting birds declined by 65.7% from 1992 to 2021 (Table 1). Declines in abundance generally reflect the imbalance between recruitment and apparent survival (Table 2). Most of the top N-mixture abundance models had similar structures (Appendix 1) with detection rates declining with advancing survey date for five of the ten species (Appendix 1). Abundance was influenced by marsh size in three of ten species (Appendix 1) and was greater in larger marshes for these species. Coefficients for predictors included in the top-ranking N-mixture models are provided in Appendix 2.

**Table 1:** Mean (±SE) abundance (λ) and t-test results for the two time periods (1992 and 2021) when marsh bird surveys were conducted at 186 survey points within the Chesapeake Bay region in Virginia, USA. Species within each group are listed beneath the group type (obligate saltmarsh, obligate marsh, and facultative marsh nesters) and community wide totals are at the bottom of table.

Species	λ 1992	λ 2021	T-test results		
			t	df	p
Obligate Salt marsh	4.46 (0.20)	2.49 (0.17)	7.53	359.38	<0.001
Clapper Rail	0.72 (0.06)	0.72 (0.06)	0.67	369.98	0.948
Willet	1.13 (0.11)	0.36 (0.06)	8.15	255.76	<0.001
Seaside Sparrow	2.12 (0.10)	1.39 (0.13)	4.80	350.23	<0.001
Obligate Marsh	3.20 (0.26)	0.59 (0.07)	9.76	210.02	<0.001
Virginia Rail	1.95 (0.24)	0.05 (0.02)	7.99	186.64	<0.001
Sedge Wren	0.08 (0.03)	0.00 (0.00)	2.83	185.00	0.005
Marsh Wren	0.65 (0.09)	0.07 (0.02)	8.21	202.80	<0.001
Red-winged Blackbird	0.53 (0.07)	0.47 (0.06)	0.65	367.85	0.517
Facultative Marsh	2.41 (0.23)	0.38 (0.05)	21.87	243.61	<0.001
Song Sparrow	0.40 (0.04)	0.01 (0.01)	8.67	196.00	<0.001
Boat-tailed Grackle	1.82 (0.23)	0.36 (0.05)	6.31	201.70	<0.001
Eastern Meadowlark	0.19 (0.03)	0.01 (0.01)	5.61	232.59	<0.001
All Species	10.08 (0.46)	3.46 (0.21)	13.08	256.64	<0.001

**Table 2:** Detection (±SE), Recruitment (±SE), and Apparent Survival (±SE) for marsh birds surveyed within the Chesapeake Bay region in Virginia, USA.

Species	Detection	Recruitment	Apparent Survival
Obligate Salt marsh			
Clapper Rail	0.25 (0.03)	0.77 (0.16)	0.27 (0.12)
Willet	0.12 (0.02)	0.82 (0.20)	0.04 (0.05)
Seaside Sparrow	0.50 (0.02)	0.59 (0.11)	0.41 (0.04)
Obligate Marsh			
Virginia Rail	0.12 (0.04)	0.45 (0.38)	0.05 (0.05)
Sedge Wren	0.26 (0.00)	0.00 (0.00)	0.00 (0.00)
Marsh Wren	0.22 (0.05)	0.09 (0.05)	0.04 (0.05)
Red-winged Blackbird	0.22 (0.03)	0.60 (0.11)	0.20 (0.08)
Facultative Marsh			
Song Sparrow	0.36 (0.04)	0.01 (0.01)	0.00 (0.00)
Boat-tailed Grackle	0.07 (0.03)	0.31 (0.15)	0.00 (0.04)
Eastern Meadowlark	0.17 (0.05)	0.02 (0.01)	0.00 (0.00)

### Obligate salt marsh nesters

Abundance has declined for obligate salt marsh nesters as a group but three obligate species have been more stable compared to most other species. The clapper rail is one of only two species that did not experience a decline and the number of occupied points was comparable between the two survey periods (89 vs 96). Occupation of marsh patches for both seaside sparrows and willets declined from 12 to 9 over the study period. Willet suffered the greatest decline in abundance 68%, though the number of occupied patches was less severe between the survey periods (57.0%). Seaside sparrows declined by 34.4% between survey periods but were more concentrated in 2021 compared to 1992. Seaside sparrows occurred within 169 plots in 1992 compared to only 99 in 2021 but peak numbers recorded per occupied plot was significantly higher (mean abundance ± SE for 1992 vs 2021 = 2.3 ± 0.10 and 2.6 ± 0.16, df = 438.75, t-statistic = -5.69, p < 0.001) in 2021 compared to 1992.

### Obligate marsh nesters

All of the obligate marsh nesters except for red-winged blackbird declined dramatically over the survey interval led by sedge wren (100%) and Virginia rail (97.4%). The sedge wren occurred within ten survey plots across three marshes in 1992 but was not detected in 2021. In 1992 this species was confined to a band of high marsh that supported scattered saltbush. Nearly all of the saltbush had been lost by 2021. The Virginia rail was widespread within low marsh areas in 1992 but very restricted by 2021. Occupied plots declined from 50 within 11 marshes to only 9 within 4 marshes. Marsh wrens showed a similar pattern with occupied plots declining from 64 to only 13. During 1992 surveys the majority of marsh wren detections were within tall cord grass. Most of these tall cord grass patches were absent in 2021 and remaining marsh wrens occupied black needlerush. The red-winged blackbird is the only marsh obligate that did not decline. Red-winged blackbirds showed a clear shift in distribution over the period. Of the 88 plots that were occupied during the two time periods, 31 (35.2%) were occupied in only 1992 and 32 (36.3%) were only occupied in 2021. During 1992 this species was primarily associated with patches of tall cordgrass but in 2021 was almost exclusively associated with patches of *Phragmites*. This shift reflects the loss of tall cordgrass and the expansion of *Phragmites* and the capacity of red-wings to exploit this shift in vegetation.

### Facultative marsh nesters

All of the facultative marsh nesters experienced catastrophic declines between the two survey years led by song sparrow (97.5%), eastern meadowlark (94.7%) and boat-tailed grackle (80.2%). The number of occupied plots declining from 64 to 2, 34–1 and 55–49 for song sparrow, eastern meadowlark and boat-tailed grackle respectively. Though boat-tailed grackle declines were less severe than the other two species, mean abundance at occupied plots declined from 6.9 ± 0.2 birds per plot to 1.2 ± 0.1 birds per plot All of these species were associated with the marsh-upland ecotone and the high marsh during both survey years. Within this study area, both song sparrows and eastern meadowlarks are ground nesters.

## Discussion

Abundance for all of the species groups and the majority of individual species declined within the survey network during the study period. The magnitude of decline was highest for facultative nesters, marsh obligates and salt marsh obligates respectively. The result of these differences is that marsh obligates have become an increasingly dominant portion of the species assemblage over time. This pattern is consistent with the prediction within salt marshes [25] and habitats in general that specialists will achieve higher densities and may persist longer than generalists as habitats are degraded [43,44]. The longer-term consequence of specialization is that the salt marsh obligates may be lost from the region if marsh degradation continues to its conclusion. Two species including the eastern black rail and the susurrans Henslow’s sparrow (*C. henslowii susurrans*) are believed to have been lost from salt marshes throughout the Chesapeake Bay during the study period [10,12]. Both of these species nested historically within the high marsh portion of salt marshes but were lost from the study area prior to the 1992 survey.

Four species including saltmarsh sparrow, sedge wren, eastern meadowlark and song sparrow were extirpated or nearly extirpated within focal marshes during the study period. The saltmarsh sparrow is confined to salt marshes, has been declining throughout its range [45], has been projected to be pushed to extinction under current sea-level rise scenarios [5] and is a candidate for federal listing. This species along with the Nelson’s sparrow (*A. nelsoni*) occurs in high densities within Chesapeake Bay marshes during the winter period [46,47] and has a late spring departure window [30]. Saltmarsh sparrows were detected within the survey network during May surveys in both 1992 and 2021. However, this species was detected within only a single point during the June and July surveys of 1992 and none were detected during the late survey rounds of 2021. A nest found within the study area in 1992 is the last nesting record known for the study area and the broader western shore of the Chesapeake Bay [33]. The sedge wren was detected within high marsh patches with scattered saltbush in 1992 but absent in 2021. The study area is near the southern range limit for breeding and the species does occur sporadically within inland nontidal marshes within the region [12]. The eastern meadowlark was widespread throughout patches of high marsh dominated by saltmeadow hay and salt grass in 1992 but only a single point in 2021. This species continues to nest within upland farm fields and grasslands within the region. Song sparrows were widespread within all focal marshes in 1992 but were detected only twice within a single marsh in 2021. Song sparrows within the study area occurred along the marsh-upland ecotone and foraged within the salt marsh. Although no attempt was made to identify birds to subspecies the behavior and habitat use is consistent with accounts of the Atlantic song sparrow (*M. m. atlantica*) that is a specialist on dunes and salt marshes of the outer Atlantic coast [48,49]. The dramatic loss of breeding song sparrows throughout the marsh network warrants further investigation regarding the status of this unique Atlantic subspecies.

Three other species including Virginia rail, marsh wren and boat-tailed grackle were notable in the magnitude of their decline. Common throughout the marsh network in 1992, Virginia rails were rare and patchy in 2021. This species primarily nests within nontidal marshes and the population trend has been positive on both regional and range-wide scales [50,51]). Similar to Virginia rail, marsh wrens also declined dramatically, nest within nontidal wetlands and have a positive trend on regional and range-wide scales [50,51]. Boat-tailed grackles experienced dramatic declines but unlike the other two species do not breed away from the coast and the population trends on both regional and range-wide scales are negative [50,51].

Clapper rails and red-winged blackbirds were the most resilient of all species examined and abundance was relatively even over the study period. Clapper rails are restricted to tidal salt marshes and select nest locations with dense overhead vegetation for concealment and that are on high points relative to the surrounding territory [52–55]. Flooding is a common cause of nest failure and nest elevation is an important determinant of success [52,55]. The slight increasing trend within the study area is consistent with the trend on both regional and range-wide scales [51]. Clapper rails within the study area were most commonly associated with patches of high spartina along creek banks and dense patches of black needlerush. Red-winged blackbirds nest within salt marshes but also nest within most wetland types throughout the region. Space use for red-winged blackbirds shifted over time with 35% and 36% of points used being exclusive to 1992 and 2021 respectively. This pattern is consistent with the shift in vegetation use observed over time. Most of the birds observed in 1992 were associated with tall spartina while the overwhelming majority of birds observed in 2021 were associated with *Phragmites australis*. As within many other regions [56–58], *Phragmites* has expanded its footprint throughout the lower Chesapeake Bay during the study period and red-winged blackbirds have benefited from this expansion.

In general, the magnitude of observed declines was greater for facultative salt marsh nesters compared to salt marsh obligates. Several morphological and life history traits have been identified for birds considered salt marsh specialists including darker dorsal plumage, salt tolerance, elevation of nests above the ground, lower investment in individual clutches (presumably allowing for multiple nesting attempts), ability of eggs to survive submersion, capability to rapidly recycle following nest loss to a flooding event [11] and breeding in higher densities [25]. These adaptations may have provided obligates with greater resilience compared to facultative species. The frequency and magnitude of tidal flooding has increased within the Chesapeake Bay [59] and around the globe [60]. Some obligate species have been shown to adjust nest height within vegetation in response to flooding risk and/or to time nesting attempts to take advantage of the gap between lunar spring tides [e.g., 61–64]. Species that are confined to nesting on the ground such as eastern meadowlark and song sparrow have no opportunity to adjust nest heights and if they are unable to time nesting relative to the lunar cycle would be effectively eliminated from marshes subjected to chronic flooding.

Although several threats to tidal marsh birds have been identified within the Chesapeake Bay [22], no attempt has been made to evaluate possible factors that may be responsible for population declines within the study area. Sea level rise SLR (and associated nest flooding, marsh loss and shifts in plant composition) and nest predation have been identified as the two dominant factors responsible for demographic stress for populations in other areas along the Atlantic Coast with their relative importance shifting site to site [e.g., 5,13,63,65]. For some species these two factors may interact since vertical adjustments in nest height to avoid flooding may increase exposure to aerial predators.

It should be noted that the results presented here are the result of only two surveys separated by a long interval. This data configuration makes it difficult to exclude the possibility that the pattern is the result of a stochastic effect. However, both the magnitude and consistency of the pattern across several species with differing ecologies makes this explanation unlikely. Also, the pattern is consistent with other results from the Chesapeake Bay [10,22].

### Management implications

Birds that have nested historically within salt marshes of the Chesapeake Bay are declining such that the habitat role that these marshes have played is diminishing. Despite their resilience over the study period, salt marsh obligates are of high conservation concern over the longer term due to their specialization on a habitat that is currently experiencing rapid change [66]. Although the underlying factors causing declines have not been fully explored within the Chesapeake Bay, the patterns are consistent with populations within other regions where SLR and nest predation are believed to be playing dominant roles [e.g., 13,64,65,67]. Both of these factors are likely to continue to be management concerns for the foreseeable future. There is consensus that SLR will continue on a global scale [68] and that the Chesapeake Bay will continue to be a SLR hotspot due to ongoing subsidence [17,18]. Predicted SLR has been estimated to cause dramatic losses to marsh area and habitat availability for salt marsh obligates over the next few decades [67,69,70]). Populations that occupy marshes that persist are predicted to face possible demographic collapse due to flooding risk alone [5]. Predation is expected to continue to exacerbate demographic stress.

How to mitigate the effects of SLR and predation on tidal marsh bird populations before they are extirpated from the Atlantic Coast is a topic of urgent interest. Flooding risk reflects the height of nests (and associated marsh elevation) and the magnitude of flooding events when nests are present. Suggestions for the amelioration of flooding risk have focused on elevating the marsh surface either by sediment deposition [71,72] or by facilitating marsh migration upslope [73,74]. Barriers to marsh migration include human-made structures, maritime forests and *Phragmites* all of which have been shown to inhibit the progress of upslope migration. Removal of these barriers within areas with appropriate slope facilitates natural marsh migration. Predation risk reflects the level of exposure of nests to searching predators and the magnitude of pressure from the predator community. Suggestions to reduce predation pressure to improve nesting success have focused on traditional predator removal [65]. Although predator removal has elevated reproductive rates within some habitats [75] this approach has not been tested within tidal salt marshes. The practicality and efficacy of executing these management options on a scale that would be relevant to populations have not been evaluated within the study area or the broader Chesapeake Bay but offer an opportunity for future study.

## Supporting information

Appendix 1 Model selection summary for N-mixture abundance models. Population parameters include abundance λ, recruitment γ, apparent survival ω, individual detection *r.*
(DOCX)

Appendix 2 Coefficients (β) for predictors included within the detection (*r*) and abundance (λ) functions of the top ranking species-specific N-mixture models. (DOCX)
