# Supplementary material for: Decline of salt marsh-nesting birds within the lower Chesapeake Bay (1992–2021)
Source: PLoS One. 2025 Jun 2;20(6):e0323254. doi: 10.1371/journal.pone.0323254 (PMC12129188; doi:10.1371/journal.pone.0323254)
Supplement: Appendix 1 — Population parameters include abundance λ, recruitment γ, apparent survival ω, individual detection r. (DOCX) [file pone.0323254.s001.docx]

Appendix 1. Model selection summary for N-mixture abundance models. Population parameters include abundance λ, recruitment γ, apparent survival ω, individual detection *r.*

| Species | Model | Distribution | K | Log-likelihood | AIC | ΔAIC | Model Weight |
| --- | --- | --- | --- | --- | --- | --- | --- |
| Willet | λ (.), γ (.), ω (.)*, r (*Survey Date) | ZIP | 6 | -812.62 | 1637.24 | 0.00 | 0.73 |
|  | λ (Marsh Size), γ (.), ω (.)*, r (*Survey Date) | ZIP | 7 | -812.62 | 1639.24 | 2.00 | 0.27 |
|  | λ (.), γ (.), ω (.)*, r (*.) | ZIP | 5 | -820.12 | 1650.23 | 12.99 | 0.00 |
|  | λ (Marsh Size), γ (.), ω (.)*, r (*.) | ZIP | 6 | -820.11 | 1652.22 | 14.98 | 0.00 |
|  | λ (.), γ (.), ω (.)*, r (*.) | P | 4 | -841.94 | 1691.88 | 54.64 | 0.00 |
| Clapper Rail | λ (.), γ (.), ω (.)*, r (*Survey Date) | P | 5 | -882.26 | 1774.52 | 0.00 | 0.61 |
|  | λ (Marsh Size), γ (.), ω (.)*, r (*Survey Date) | P | 6 | -881.72 | 1775.44 | 0.92 | 0.39 |
|  | λ (.), γ (.), ω (.)*, r (*.) | P | 4 | -901.43 | 1810.86 | 36.34 | 0.00 |
|  | λ (Marsh Size), γ (.), ω (.)*, r (*.) | P | 5 | -900.75 | 1811.49 | 36.97 | 0.00 |
|  | λ (.), γ (.), ω (.)*, r (*.) | ZIP | 5 | -901.44 | 1812.87 | 38.35 | 0.00 |
| Virginia Rail | λ (.), γ (.), ω (.)*, r (*Survey Date) | ZIP | 6 | -298.01 | 608.01 | 0.00 | 0.66 |
|  | λ (Marsh Size), γ (.), ω (.)*, r (*Survey Date) | ZIP | 7 | -297.70 | 609.39 | 1.38 | 0.33 |
|  | λ (.), γ (.), ω (.)*, r (*.) | ZIP | 5 | -304.00 | 618.00 | 10.00 | 0.00 |
|  | λ (Marsh Size), γ (.), ω (.)*, r (*.) | ZIP | 6 | -303.51 | 619.02 | 11.01 | 0.00 |
|  | λ (.), γ (.), ω (.)*, r (*.) | P | 4 | -309.62 | 627.23 | 19.22 | 0.00 |
| Marsh Wren | λ (Marsh Size), γ (.), ω (.)*, r* (.) | ZIP | 6 | -376.29 | 764.58 | 0.00 | 0.98 |
|  | λ (Marsh Size), γ (.), ω (.)*, r* (Survey Date) | ZIP | 7 | -379.10 | 772.19 | 7.61 | 0.03 |
|  | λ (.), γ (.), ω (.)*, r* (.) | ZIP | 5 | -402.73 | 815.45 | 50.88 | 0.00 |
|  | λ (.), γ (.), ω (.)*, r* (Survey Date) | ZIP | 6 | -402.73 | 817.45 | 52.88 | 0.00 |
|  | λ (.), γ (.), ω (.)*, r (*.) | P | 4 | -407.78 | 823.55 | 58.97 | 0.00 |
|  | λ (Marsh Size), γ (.), ω (.)*, r* (Survey Date) | ZIP | 7 | -66.77 | 147.54 | 0.00 | 0.58 |
| Sedge Wren | λ (Marsh Size), γ (.), ω (.)*, r* (.) | ZIP | 6 | -68.09 | 148.17 | 0.63 | 0.42 |
|  | λ (.), γ (.), ω (.)*, r* (Survey Date) | ZIP | 6 | -77.73 | 167.45 | 19.91 | 0.00 |
|  | λ (.), γ (.), ω (.)*, r* (.) | ZIP | 5 | -79.17 | 168.33 | 20.79 | 0.00 |
|  | λ (.), γ (.), ω (.)*, r (*.) | P | 4 | -89.84 | 183.68 | 36.14 | 0.00 |
| Seaside Sparrow | λ (Marsh Size), γ (.), ω (.)*, r* (Survey Date) | P | 6 | -1660.01 | 3332.01 | 0.00 | 0.52 |
|  | λ (Marsh Size), γ (.), ω (.)*, r* (.) | P | 5 | -1661.10 | 3332.19 | 0.18 | 0.48 |
|  | λ (.), γ (.), ω (.)*, r* (Survey Date) | P | 5 | -1669.24 | 3348.48 | 16.48 | 0.00 |
|  | λ (.), γ (.), ω (.)*, r* (.) | P | 4 | -1670.37 | 3348.74 | 16.73 | 0.00 |
|  | λ (.), γ (.), ω (.)*, r* (.) | ZIP | 5 | -1670.37 | 3350.74 | 18.74 | 0.00 |
| Song Sparrow | λ (.), γ (.), ω (.)*, r* (.) | P | 4 | -343.33 | 694.65 | 0.00 | 0.43 |
|  | λ (Marsh Size), γ (.), ω (.)*, r (*.) | P | 5 | -343.25 | 696.50 | 1.84 | 0.17 |
|  | λ (.), γ (.), ω (.)*, r* (Survey Date) | P | 5 | -343.26 | 696.51 | 1.85 | 0.17 |
|  | λ (.), γ (.), ω (.)*, r (*.) | ZIP | 5 | -343.27 | 696.53 | 1.88 | 0.17 |
|  | λ (Marsh Size), γ (.), ω (.)*, r* (Survey Date) | P | 6 | -343.17 | 698.34 | 3.69 | 0.07 |
| Eastern Meadowlark | λ (.), γ (.), ω (.)*, r* (Survey Date) | P | 5 | -183.14 | 376.28 | 0.00 | 0.66 |
|  | λ (Marsh Size), γ (.), ω (.)*, r* (Survey Date) | P | 6 | -182.85 | 377.69 | 1.41 | 0.33 |
|  | λ (.), γ (.), ω (.)*, r (*.) | P | 4 | -189.31 | 386.61 | 10.33 | 0.00 |
|  | λ (.), γ (.), ω (.)*, r (*.) | ZIP | 5 | -188.84 | 387.68 | 11.40 | 0.00 |
|  | λ (Marsh Size), γ (.), ω (.)*, r* (.) | P | 5 | -189.02 | 388.04 | 11.76 | 0.00 |
| Red-winged Blackbird | λ (Marsh Size), γ (.), ω (.)*, r* (Survey Date) | ZIP | 7 | -691.12 | 1396.23 | 0.00 | 0.60 |
|  | λ (.), γ (.), ω (.)*, r (*Survey Date) | ZIP | 6 | -692.69 | 1397.38 | 1.15 | 0.34 |
|  | λ (Marsh Size), γ (.), ω (.)*, r (*.) | ZIP | 6 | -694.77 | 1401.53 | 5.30 | 0.04 |
|  | λ (.), γ (.), ω (.)*, r (*.) | ZIP | 5 | -696.23 | 1402.45 | 6.22 | 0.03 |
|  | λ (.), γ (.), ω (.)*, r (*.) | P | 4 | -717.10 | 1442.20 | 45.97 | 0.00 |
| Boat-tailed Grackle | λ (.), γ (.), ω (.)*, r* (.) | ZIP | 5 | -374.56 | 759.12 | 0.00 | 0.45 |
|  | λ (.), γ (.), ω (.)*, r* (Survey Date) | ZIP | 6 | -374.16 | 760.32 | 1.20 | 0.25 |
|  | λ (Marsh Size), γ (.), ω (.)*, r* (.) | ZIP | 6 | -374.38 | 760.76 | 1.65 | 0.20 |
|  | λ (Marsh Size), γ (.), ω (.)*, r* (Survey Date) | ZIP | 7 | -373.95 | 761.90 | 2.79 | 0.11 |
|  | λ (.), γ (.), ω (.)*, r (*.) | P | 4 | -400.25 | 808.49 | 49.37 | 0.00 |
