# Supplementary material for: Decline of salt marsh-nesting birds within the lower Chesapeake Bay (1992–2021)
Source: PLoS One. 2025 Jun 2;20(6):e0323254. doi: 10.1371/journal.pone.0323254 (PMC12129188; doi:10.1371/journal.pone.0323254)
Supplement: Appendix 2 — (DOCX) [file pone.0323254.s002.docx]

Appendix 2. Coefficients (β) for predictors included within the detection (*r*) and abundance (λ) functions of the top ranking species-specific N-mixture models.

| Species | Function | Predictor | β | p-value |
| --- | --- | --- | --- | --- |
| Clapper Rail | *r* | Day of Year | -0.37±0.06 | <0.001 |
| Willet | *r* | Day of Year | -0.23±0.06 | <0.001 |
| Virginia Rail | *r* | Day of Year | -0.02±0.01 | 0.001 |
| Eastern Meadowlark | *r* | Day of Year | -0.02±0.01 | 0.002 |
| Red-winged Blackbird | *r* | Day of Year | -0.20±0.07 | 0.006 |
| Seaside Sparrow | λ | Marsh Size | 0.21±0.05 | <0.001 |
| Sedge Wren | λ | Marsh Size | 0.02±0.01 | 0.005 |
| Marsh Wren | λ | Marsh Size | 0.77±0.09 | <0.001 |
